# A Comparative Analysis of the Occluding Effects on Dentinal Tubules With the Use of Er:YAG Laser and a Commercially Available Desensitizing Agent: An In Vitro Scanning Electron Microscopic Study

**DOI:** 10.7759/cureus.43791

**Published:** 2023-08-20

**Authors:** Lynn Johnson, Abhishek Soni, Satish Kaliappan, Supriya Mishra, Laxmi Kaushal, Sakshi Teware

**Affiliations:** 1 Department of Periodontology, Rama Dental College, Kanpur, IND; 2 Department of Periodontology, Maitri College of Dentistry and Research Centre, Durg, IND; 3 Department of Periodontology, Government Dental College and Hospital, Raipur, IND; 4 Department of Periodontology, RR Kambe Dental College and Hospital, Akola, IND

**Keywords:** scanning electron microscope, desensitizing tooth-paste, dentinal tubule occlusion, laser, dentinal hypersensitivity

## Abstract

Objective

This in vitro scanning electron microscopic (SEM) study aimed to compare the effect of Er:YAG laser and a commercially launched dental product namely 8% arginine-calcium carbonate on exposed dentinal tubules.

Materials and methods

A total of 120 tooth samples prepared from healthy maxillary first premolars extracted due to orthodontic reasons were grouped randomly into four groups of 30 samples each - Group I: control group (C); Group II: laser group (LG); Group III: toothpaste group (TP) and Group IV: laser + toothpaste group (LT). The samples in Group II-IV were treated with the respective test agents and were placed under SEM to study the changes in the dentinal tubule number and diameter. The data obtained from SEM were then subjected to statistical analysis using an unpaired t-test.

Results

The unpaired t-test revealed extreme statistical differences in means between the test and the control groups and among the test groups (p<0.0001).* *The results we obtained within the scope of this study showed that both the Er:YAG laser (1.3 W, 100 mJ, 3 Hz, 60 s twice) and dentifrice containing 8% arginine-calcium carbonate as the main ingredient can significantly reduce the number and diameter of the open dentinal tubules.

Conclusion

Our findings have demonstrated that both the 8% arginine-calcium carbonate technology and Er:YAG laser successfully reduced the number and diameter of the open dentinal tubules and hence can be promising agents to deal with dentinal hypersensitivity in future clinical studies.

## Introduction

Dentine hypersensitivity, as defined by the nternational workshop and later modified by Canadian Advisory Board (2003), is defined as a short, sharp pain arising from exposed dentine in response to stimuli, typically thermal, evaporative, tactile, osmotic or chemical, and which cannot be ascribed to any other dental defect or disease (previous pathology). The condition is prevalent in individuals between 20 and 40 years of age, with buccocervical areas of the permanent teeth being most affected. Intra-orally, canines and premolars are the most affected followed by incisors and second premolars and molars [1].

The hydrodynamic theory states that when exposed dentinal tubules are intrigued by changes in the osmotic pressure and temperature, fluid in the tubule is displaced, which is then turned over to the nerve fibers of the pulp, causing stimulation that is propagated as pain [2,3]. Thus, a logical approach to control pain emerging from the exposed tooth dentin would thus be to reduce or block the diameter of the dentinal tubules [4]. Lasers are considered effective to reduce dentinal hypersensitivity because of their analgesic effect on pulpal nerves, sealing effect on dentinal tubules, or placebo effect. Various soft and hard tissue lasers have been used in dental clinics with varying results. However, the at-home classical approach to reduce tooth sensitivity has been the application of desensitizing dentifrice basically containing either potassium ions to depolarize or inactivate the nerves, blocking the sensation of pain, or the use of occlusion technology such as oxalates, stannous or strontium precipitates, amorphous calcium phosphate, bioactive glass, and composite resin to plug or seal the tubules to prevent fluid movement within the dentinal tubules and the subsequent pain response [1].

A recently launched dentifrice containing arginine and calcium carbonate has been available in the market for treating dentin hypersensitivity. The product is unique in that it has arginine and calcium; its two main components occur naturally in saliva and these two work collectively to increase the natural mechanism of occlusion by depositing a dentin-like mineral containing calcium-phosphate inside the tubules and also act as a protective layer on dentin surface [5].

Various studies have been conducted in the past using lasers in combination with different dentifrice, mouthwashes, and chemical agents for reducing dentinal hypersensitivity [6-8]. In this study, our objective was to analyze the occluding effects of dentinal tubules by using Er:YAG lasers and a commercially available desensitizing toothpaste containing 8% arginine-calcium carbonate and their combined effect on dentinal tubule occlusion. Hence, the aim was to evaluate the occluding effects on dentinal tubules in terms of reduction in the tubule number and diameter by a hard tissue laser (Er:YAG Laser) and a commercially available desensitizing toothpaste (8% arginine-calcium carbonate as the active ingredient) and a combination of these agents.

## Materials and methods

Sample collection

Healthy maxillary first premolars, extracted for orthodontic purposes from individuals aged 14-25 years, were collected and immersed in distilled water at room temperature. The water was changed on a weekly basis to prevent the growth of microbes. Following this, a meticulous process involving ultrasonic scaling and root planning was carried out for 10 minutes each, resulting in the exposure of the dentinal layer. Subsequently, a 2 x 2 x 1-mm section was obtained from just below the junction between the enamel and cementum on the facial side of each tooth. This section was acquired by using a double diamond disk with the assistance of water coolant. A total of 120 specimens were procured and then subjected to a series of treatments. They were initially placed in a 17% aqueous solution of ethylenediaminetetraacetic acid (EDTA) (Dent Wash, Prime Dental Products Pvt. Ltd., Thane, India). EDTA was utilized to eliminate the smear layer, thereby revealing the dentinal tubules. Following this step, the specimens were exposed to a 5% sodium hypochlorite solution (Hyposol, Prevest DenPro Limited, Jammu, India) for a duration of five minutes each. Subsequently, the specimens were thoroughly rinsed with distilled water and stored in it until further use.

Finally, 120 specimens were divided randomly into four groups of 30 samples each, as follows - Group I: control group (C), Group II: laser group (LG), Group III: toothpaste group (TP), and Group IV: laser + toothpaste group (LT). To homogenize the fluoride content in all three test groups, samples of Group II were treated with fluoride-containing toothpaste, and Groups III and IV were tested with a dentifrice that contained fluoride. Thus, the active ingredient in the study would be the Er:YAG laser and the 8.0% arginine-calcium carbonate. The Er:YAG laser part of this study was performed at Confident Dental Care, Bengaluru, Karnataka.

Group I, referred to as the control group (C), consisted of 30 samples that remained untreated with both toothpaste and laser. This group served as the baseline for comparison. These samples were examined under a scanning electron microscope (SEM) to determine the total count of open dentinal tubules and their respective diameters, serving as a reference point for the other three test groups (LG, TP, and LT). In Group II, the laser group (LG), 30 samples were exposed to a hard-tissue Er:YAG laser ( LiteTouch™, Syneorn Dental Lasers, North District, Israel; version 1.26). The laser was operated at a power setting of 1.30 watts, with an energy of 100 mJ and a frequency of 3 Hz, applied for 60 seconds twice. A water coolant was used in continuous mode [9], and the Er:YAG laser was utilized in scanning movements. It was defocused and positioned perpendicularly at a distance of 6 mm from the surface [10,11]. Group III, the toothpaste group (TP), comprised 30 samples that were treated with a specialized desensitizing toothpaste containing 8.0% arginine, calcium carbonate, and 1450 ppm fluoride (Colgate Pro-relief, Colgate, New York, NY). A small amount of this toothpaste, equivalent to the size of a pea, mixed with 1 ml of artificial saliva (Wet Mouth, ICPA Health Products Ltd, Mumbai, India), was manually applied to the facial aspect of the prepared samples by using a Camel hairbrush. The application lasted for one minute and was followed by rinsing with distilled water. In Group IV, the laser + toothpaste group (LT), a combination approach was employed. This group used a desensitizing toothpaste containing 8% arginine, calcium carbonate, and fluoride (1450 ppm), along with the utilization of the Er:YAG laser. The application procedure remained consistent with the methods described above. The toothpaste was applied first, followed by the application of the laser. It is noteworthy that the purpose of Group IV was to explore the cumulative effects of both the desensitizing toothpaste and the Er:YAG laser on the diameter and count of dentinal tubules. The identical methods of toothpaste and laser application were maintained as detailed previously.

Subsequently, the collected samples underwent a preservation process. They were initially immersed in a solution of 2.5% glutaraldehyde (Cidex, Johnson & Johnson India, Mumbai, India) for a duration of 12 hours at a temperature of 4 °C. This immersion served to effectively conserve the samples and stabilize the dentinal tubules prior to their examination through SEM [12]. Following this preservation step, a chemical dehydration procedure was carried out. This involved subjecting the samples to increasing concentrations of ethanol (25%, 50%, 70%, 90%, and 100%) for a span of five minutes each. Subsequent to this, the samples were allowed to air-dry at room temperature for a period of 24 hours.

The processed samples were subjected to further steps for analysis. They were subjected to sputter coating with a thin layer of gold. After this, the samples were meticulously observed under an SEM (Leica S440i, Leica Cambridge, Ltd. at CPRI, Bengaluru) at a voltage of 15 kilovolts. Microphotographs in black and white were acquired for each sample at different magnifications: 500x, 1000x, and 2000x. Through the use of the Leica Image Analysis Software, the subsequent analysis was conducted. This included assessing the number of tubules that were partially or completely unblocked, as well as measuring the diameter of the tubules. These measurements were obtained for all four groups under study.

Sample analysis

**Figure 1:**
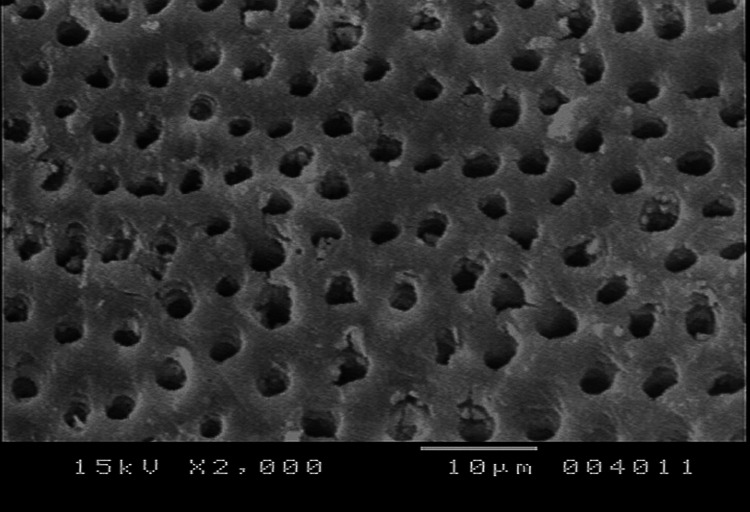
SEM photograph of the control group SEM: scanning electron microscope

**Figure 2:**
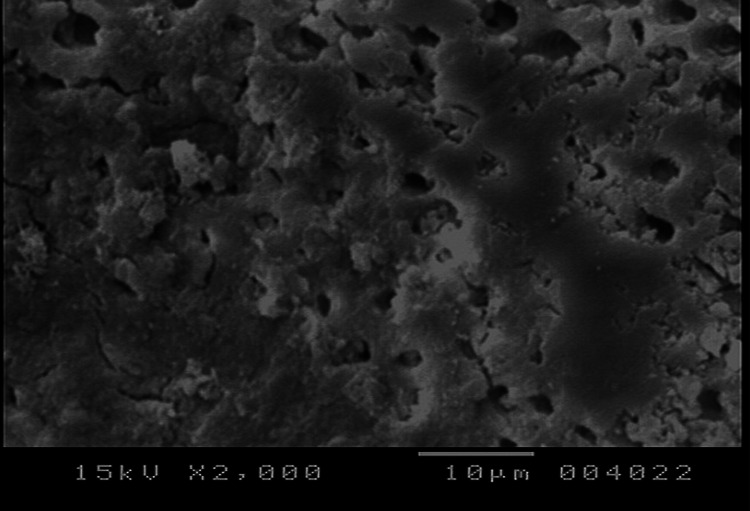
SEM photograph of the toothpaste group SEM: scanning electron microscope

**Figure 3:**
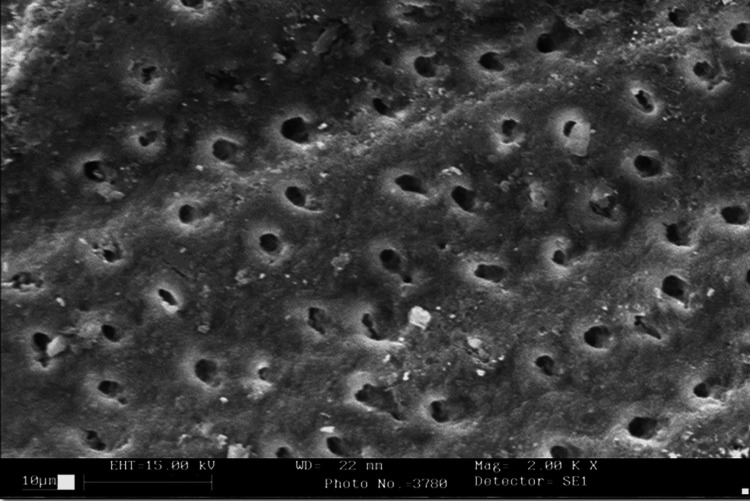
SEM photograph of the laser group SEM: scanning electron microscope

Each sample of the entire four groups was assessed for the mean tubule diameter. In the control group, each electron microphotograph taken at 2000x magnification was divided into five equal parts, and, randomly, a tubule was chosen from each part. The mean tubule diameter (in microns) for each microphotograph was obtained and thus for the entire control group, and the data were documented on a Microsoft Excel sheet. This mean tubule diameter of the control group represented the opened tubule diameter at 2000x magnification, which acted as the reference value for the test groups. Next, the three test groups were studied for the reduction in the mean tubule diameter following the same criterion as above and tabulated on the datasheet. The mean reduction in the tubule diameter in the three test groups was calculated by subtracting the mean tubule diameter from the reference value (obtained from the control group). Thus, the mean tubule diameter reduction in the three test groups was obtained. This was followed by calculation and tabulation of the mean percentage reduction of the tubule diameter, and statistical analysis using the unpaired t-test was applied for pair-wise comparison of the groups (Figures 1, 2, 3).

Using the Leica Image Analysis Software, every microphotograph in the control group was carefully assessed, and each dentinal tubule was tallied. These values were meticulously recorded and organized in a tabular format. As such, the average number of tubules present per sample under 2000x magnification was taken as the reference point [8,12].

In a similar manner, the microphotographs of the three test groups were meticulously examined to identify and count the remaining partially and completely unblocked dentinal tubules. To quantify the reduction in tubule count within the test groups, the mean number of tubules in each test group was subtracted from the mean number of control group tubules. These values were systematically tabulated, and subsequently, the average reduction in the number of tubules was computed for each group.

The ensuing step involved the calculation and tabulation of the mean percentage reduction in the number of dentinal tubules, employing the same methodology outlined above. For the purpose of statistical evaluation, the unpaired t-test was employed, enabling a pairwise comparison of the different groups.

## Results

The mean tubule diameter for the control group was 1.97 ± 0.49 µm (i.e., 0% reduction). Mean tubule diameter reduction achieved in Er: YAG laser-treated group was 0.95 ± 0.33 µm (46.71%); it was 0.18 ± 0.25 µm (40.23%) in the toothpaste group and 0.73 ± 0.13 µm (37%) in the laser + toothpaste group. The unpaired t-test revealed extreme statistical differences in means between the test and the control groups and among the test groups (p<0.0001). Thus, all three test samples showed a significant reduction in the mean tubule diameter as compared to the control group (Tables 1, 2).

**Table 1:** Dentinal tubule diameter reduction (in microns and in percentage) in the 3 test groups with reference to the control group

Variables	Control group (C)	Laser group (LG)		Toothpaste group (TP)		Laser + toothpaste group (LT)	
	Diameter of open dentinal tubule	Reduction obtained (in microns)	Reduction obtained (in %)	Reduction obtained (in microns)	Reduction obtained (in %)	Reduction obtained (in microns)	Reduction obtained (in %)
Mean	1.97	0.95	48.2	0.83	42.1	0.78	39.6
Standard deviation (SD)	0.49	0.33	17.3	0.23	11.8	0.13	6.7

**Table 2:** Unpaired t-test for pairwise comparison of the groups for tubule diameter reduction obtained in the test groups with reference to the control group

Variables	Mean	SD	P-value
Control	0	0	
Control vs. laser	0.95	0.33	<0.001
Control vs. paste	0.83	0.23	<0.001
Control vs. laser + paste	0.78	0.13	<0.001
Laser	0.95	0.33	0.0111
Laser + paste	0.78	0.13	
Laser	0.95	0.33	0.1077
Paste	0.83	0.23	
Paste	0.83	0.23	0.0426
Laser + paste	0.78	0.13	

The mean number of dentinal tubules observed in the control group was 47.07 ± 15.23 (i.e., 0% blocked). The number of tubules blocked in the laser group was 28.6 ± 10.58 (60.96%); it was 34.43 ± 9.22 (72.27%) in the toothpaste group and 16.4 ± 11.79 (33.61%) in the laser + toothpaste group. There was an extreme statistical difference in the mean number of tubules blocked in the three test groups with reference to the control group, as well as in the laser + toothpaste group in comparison to the laser and paste group (p<0.0001). The comparison of the laser and the paste groups also showed a significant difference in the mean number of tubules (p=0.0266) (Tables 3, 4).

**Table 3:** Number and percentage of dentinal tubules blocked in the 3 test groups with reference to the control group

Variables	Control group (C)	Laser group (LG)		Toothpaste group (TP)		Laser + toothpaste group (LT)	
	Total number of open tubules	Number of tubules blocked	Percentage of tubules blocked	Number of tubules blocked	Percentage of tubules blocked	Number of tubules blocked	Percentage of tubules blocked
Mean	47	28.6	60.9	34.4	74	16.4	33.5
Standard deviation (SD)	15.2	10.6	22.5	9.2	19.3	11.8	23.3

**Table 4:** Unpaired t-test for pairwise comparison of the groups to statistically evaluate the number of dentinal tubules blocked in the test groups with reference to the control group SD: standard deviation

Variables	Mean	SD	P-value
Control	47.07	15.23	
Control vs. laser	28.6	10.58	0.0001
Control vs. paste	34.43	9.22	0.0003
Control vs. laser + paste	16.4	11.79	<0.001
Laser	28.6	10.58	0.0266
Paste	34.43	9.22	
Paste	34.43	9.22	0.0001
Laser + paste	16.4	11.79	
Laser	28.6	10.58	<0.0001
Laser + paste	16.4	11.79	

Thus, it was observed that both the test agents, Er:YAG laser and the 8% arginine-calcium carbonate dentifrice, and their combination were effective in reducing the number and diameter of the dentinal tubules. But the combination of the laser and the toothpaste was no better than the two test products used alone.

## Discussion

The parameters of Er:YAG laser considered in this study were the same as those analyzed by Birang et al. in their study [9]. They compared the impact of Er:YAG laser (100mJ, 3Hz, 60s twice) and Nd:YAG (1W, 15Hz, 60s twice) laser on dentinal hypersensitivity treatment in 63 patients and reported that both the lasers have an acceptable therapeutic effect [9]. Schwartz et al. (2002), the first researchers to try Er:YAG laser for dentin hypersensitivity, found that high absorption of the Er:YAG laser emission wavelength in water resulted in the evaporation of dentinal fluid from tubules and smear layer, especially within the first six months. Additionally, the probable anti-bacterial feature of the laser may be also bestowed to the desensitizing effects [11]. Similarly, various other authors such as Zhuang et al. [12], Ehlers et al. [13], Yu et al. [14], and Badran et al. [15] have reported the use of this laser for reducing hypersensitivity. Zhuang et al. [12], in their in vitro study, analyzed the effects of the Er:YAG laser with different parameter settings on the occlusion of dentinal tubules (DT) and concluded that it is a suitable parameter to occlude open tubules. Badran et al. [15] also explored in vitro the microscopical occluding effects of Er:YAG laser on exposed dentinal tubules.

The desensitizing dentifrice containing 8% arginine-calcium carbonate and fluoride (1450 ppm) as monofluorophosphate, which was used in this given study, was efficient in reducing the diameter and number of tubules. The results of this study are similar to those of several SEM studies, such as those by Petrou et al. (2009) [5], Cummins et al. [16], and Lavender et al. [17]. The combination of arginine and calcium in alkaline pH appears as the key component in determining the effectiveness of occlusion. In two similar eight-week studies, one in Canada by Ayad et al. [18] and the other in Italy by Docimo et al. [19], 8% arginine toothpaste provided a statistically significant reduction of dentin hypersensitivity in response to tactile and air blast measures compared to Sensodyne Total Care F at two, four, and eight weeks, respectively. Barlow et al. also did a comparison of two marketed desensitizing dentifrice, one containing 50000 ppm KNO3 and 1450 ppm fluoride as NaF and the other with 80000 ppm arginine, bicarbonate, calcium carbonate, and 1450 ppm fluorine as NaMFP with a different mode of action in a randomized, eight-week, longitudinal clinical study; they concluded that both treatment groups displayed a significant decrease in sensitivity at one, two, and four weeks [20].

In a study comparing the impact of bioactive glass and arginine dentifrices on the reduction of dentin permeability and acid tolerance, Champaiboon et al. concluded that arginine demonstrated the highest reduction in dentin permeability (39.26%) compared to bioactive glass (32.27%) and the control group (21.71%) [21]. The SEM results observed by Uraz et al. showed effective tubule occlusion in tooth specimens treated with 8% arginine-CaCO_3_ compared to 1.23% NaFgel [22]. To our knowledge, this is the first study comparing the effect of arginine technology and hard tissue Er:YAG laser individually and in combination, to study its effect on dentine tubules. Since no similar studies have been reported in the literature, the results of the present study could not be compared to other studies.

In the in vitro investigation by Al-Saud and Al-Nahedh [23], the researchers aimed to microscopically assess and compare the occluding effects of the Nd:YAG laser and various dentin desensitizing agents on human dentinal tubules. The study employed 64 extracted intact human molars, wherein each dentin surface underwent treatment or served as a control. Utilizing EDTA to create open dentinal tubules, the Nd:YAG laser and four dentin desensitizers (Gluma® desensitizer, Tenure Quick®, Quell™ desensitizer, and VivaSens®) were investigated. The laser-treated samples exhibited altered dentinal structure through reduction or complete obliteration of tubule lumens, with the two-minute laser group displaying bubble-like changes. Statistically, the two-minute group displayed higher partially or fully occluded tubules compared to the one-minute group. All desensitizing agents led to tubule occlusion, yet variations in precipitate appearance, coverage level, and occlusion degree were observed among the agents. Ultimately, both the Nd:YAG laser and the tested desensitizing agents achieved successful occlusion or narrowing of dentinal tubules within the study's specified timeframe.

In the study conducted by Dixit et al. [24], the objective was to assess and compare the efficacy of three different commercially available desensitizing products in occluding dentinal tubules using SEM. Ninety non-diseased human mandibular premolar teeth were utilized, with each tooth's dentinal surface exposed through abrasive polishing. The teeth were divided into three groups: Group I was treated with Admira Protect, Group II with MI paste, and Group III with Remin Pro. SEM images were taken at 2000× magnification to evaluate dentinal tubule occlusion. The results revealed that MI paste exhibited the highest dentinal tubular occlusion (2.746 ± 0.530), followed by Admira Protect (3.498 ± 0.202) and Remin Pro (4.594 ± 0.364). Statistically significant differences were observed among the desensitizing materials used. The study concluded that despite varying chemical compositions and application techniques, all three desensitizing agents effectively sealed dentinal tubules. Notably, MI paste displayed the greatest occluding capacity among the agents. This research holds clinical significance for addressing dentin hypersensitivity by identifying the most effective commercial product for occluding dentinal tubules.

Our recent understanding of the cause of dentine hypersensitivity has led to the need of designing new treatment options that will target the underlying etiology, along with treating the symptoms. It has been repeatedly implied that future research should focus on finding new materials that can yield more mechanically and chemically resistant dentine surfaces. Notably, it has been proposed that resistance to wear can be achieved by increasing the surface mineral density, whereas sealing or plugging exposed tubules with calcium and phosphate-containing dentin-like substances would increase wear resistance and acid resistance.

## Conclusions

Within the scope of the given parameters, Er:YAG laser, the 8% arginine-calcium carbonate dentifrice, and their combination were effective in reducing the diameter and number of dentinal tubules. The results obtained by the combined use of Er:YAG laser and the dentifrice were not superior to those from test agents used alone. Further in vitro, experimental, and clinical studies including larger samples/subjects can be performed to evaluate the long-term validity of the positive results obtained with these agents.
